# Cystic Echinococcosis in Hospitalized Children and Adults from Western Romania: 2007–2022

**DOI:** 10.3390/microorganisms13051035

**Published:** 2025-04-30

**Authors:** Ana Alexandra Ardelean, Maria Alina Lupu, Laurentiu Vasile Sima, Gabriel Veniamin Cozma, Alexandru Nesiu, Alin Gabriel Mihu, Octavian Marius Cretu, Tudor Rares Olariu

**Affiliations:** 1 Discipline of Parasitology, Department of Infectious Diseases, Victor Babes University of Medicine and Pharmacy, 300041 Timisoara, Romania; paduraru.ana@umft.ro (A.A.A.); lupu.alina@umft.ro (M.A.L.); 2 Center for Diagnosis and Study of Parasitic Diseases, Department of Infectious Disease, Victor Babes University of Medicine and Pharmacy, 300041 Timisoara, Romania; alin.mihu@umft.ro; 3 Patogen Preventia, 300124 Timisoara, Romania; 4 Clinical Laboratory, Municipal Clinical Emergency Hospital, 300254 Timisoara, Romania; 5 Clinical Laboratory, Institute of Cardiovascular Diseases, 300310 Timisoara, Romania; 6 Discipline of Surgical Semiology I, Department IX-Surgery I, Victor Babes University of Medicine and Pharmacy, 300041 Timisoara, Romania; sima.laurentiu@umft.ro; 7 General Surgery Clinic, Municipal Clinical Emergency Hospital, 300254 Timisoara, Romania; 8 Center for Hepato-Biliary-Pancreatic Surgery (CHBP), Victor Babes University of Medicine and Pharmacy, 300041 Timisoara, Romania; 9 Discipline of Surgical Semiology I and Thoracic Surgery, Department IX-Surgery I, Victor Babes University of Medicine and Pharmacy, 300041 Timisoara, Romania; gabriel.cozma@umft.ro; 10 Thoracic Surgery Clinic, Municipal Clinical Emergency Hospital, 300254 Timisoara, Romania; 11 Thoracic Surgery Research Center, Victor Babes University of Medicine and Pharmacy, 300041 Timisoara, Romania; 12 Department of Biology and Life Science, Faculty of Medicine, Vasile Goldis Western University of Arad, 310025 Arad, Romania; alexnesiu@yahoo.com; 13 “Aurel Ardelean” Institute of Life Sciences, Vasile Goldis Western University of Arad, 310414 Arad, Romania

**Keywords:** epidemiology, hydatid disease, cystic echinococcosis, Romania, retrospective study

## Abstract

Cystic echinococcosis (CE) is a serious health problem in many countries worldwide, including in Romania, because of the high infection rates in both humans and animals. We retrospectively assessed the demographic and epidemiological features of CE in children and adults hospitalized in Western Romania between 2007–2022. Analyzed data were collected from the hospitals’ medical records. This research involved 426 subjects (3–90 years): 60 (14.1%) children and 366 (85.9%) adults. A decreasing trend in the number of cases was noted during the analyzed period (*p* = 0.004). Multiple-organ involvement was reported in 16.7% of the children and in 6.3% of the adults (*p* = 0.005). The liver was the most commonly affected organ. The rate of lung involvement was higher in children (25%) than in adults (13.1%) (*p* = 0.02). Most of the patients had one hospital presentation (74.9%). Multiple hospitalizations were reported in 40% of the children and 22.7% of the adults (*p* = 0.004). CE is a severe zoonotic disease that impacts individuals of all ages. Despite the decline in cases, CE remains a public-health problem in Western Romania. Health programs that target risk factors and control measures should be implemented to stop the parasite’s spread and maintain the trend toward reduced numbers of CE cases.

## 1. Introduction

Cystic echinococcosis (CE), the world’s second-most-common foodborne parasitic infection, is caused by the larval stages of tapeworm *Echinococcus granulosus sensu lato* [1,2]. The disease is included in the portfolio of the World Health Organization, who prioritize its control [2].

The life cycle of *E. granulosus* depends on the interaction between predators and prey [3]. Canids are the definitive hosts of *E. granulosus* and acquire the infection when they consume viscera containing viable hydatid cysts (metacestodes). Goats, sheep, cattle, pigs, and other livestock animals represent the intermediate hosts of the parasite and harbor the parasitic metacestode in their organs [2,4,5]. Humans are considered accidental hosts for *E. granulosus*. They become infected by ingesting the parasitic eggs but are not engaged in further transmission of the infection [4,6]. Following ingestion, the embryo hatches and penetrates the wall of the small intestine. In about 70% of the cases, the embryos of *E. granulosus* remain trapped in the liver and develop into hydatid cysts (metacestodes). Smaller embryos have the ability to cross the hepatic sinusoids and enter the lungs through the right side of the heart [5,6,7]. The spleen, heart, brain, kidneys, peritoneum, and bones are among the organs that can also be affected [8,9].

Hydatid cysts (metacestodes) grow slowly. Therefore, patients are often asymptomatic for long periods of time. Usually, months or even years pass before they are diagnosed [5]. Clinical signs and symptoms depend on the organ involved [10,11]. A wide range of clinical manifestations can be reported, from clinically quiet cysts [12] to anaphylactic shock brought on by cyst rupture [2,13].

Cystic echinococcosis can be diagnosed using imaging techniques such as ultrasonography, radiography, computed tomography, or magnetic resonance imaging [4,5]. Ultrasonography has been a popular method for the detection of hydatid cysts. Diagnostic accuracy of ultrasound can approach 90%, depending on the user’s skill and technique. The cyst wall often appears as a hypoechoic layer with an echogenic line on each side. A simple, unilocular, cyst without an inner structure is usually seen. Nevertheless, the hydatid cyst frequently contains many punctate echogenic foci, which are only visible when the subject is repositioned. These foci consist of fluid and protoscolices called hydatid sand [14,15]. It is also possible for the endocyst to separate from the pericyst; as a result, “floating membranes” can be seen inside the cystic cavity [14,15].

Although it is an excellent tool for early diagnosis of CE, there are a number of reasons why ultrasonography might fail, such as obesity, excessive gas in the colon, or previous surgical procedures. With a sensitivity of about 94%, CT is essential for identifying complications during the perioperative phase [15]. Furthermore, MRI can better visualize liquid regions inside the matrix than CT can, and it is recommended that it be used whenever possible [16].

Infection with *Echinococcus* causes an antibody response; this response is primarily IgG, followed by IgM, IgA, and IgE. However, in 30–40% of cases, no antibodies are detected [15]. Over time, several serological tests for detecting IgG, IgM, and IgE antibodies have been developed. Enzyme-linked immunosorbent assays (ELISA), immunoelectrophoresis, and immunoblots (IB) are currently available antibody-detection methods that use the hydatid fluid fraction, along with native and recombinant antibodies [15]. ELISA is mostly used as a screening method, while IB is used as a confirmatory method due to its higher specificity and sensitivity [16].

The treatment and management of CE depends on multiple factors like extent of organ involvement, number of cysts, existence of fistulas, and occurrence of cyst infection or hemorrhages [15]. In general, surgery is the treatment of choice. Chemotherapy, the “watch-and-wait” strategy, percutaneous drainage combined with administration of praziquantel–albendazole, and the minimally invasive puncture-aspiration-injection-re-aspiration (PAIR) method have been used as alternative treatments [4,11,17].

Recurrence is still a major problem in the treatment of hydatid disease, even with advancements in surgical methods and chemotherapy. In CE, recurrence rates range from 0% to 22% and are observed any time from three months to twenty years following the first procedure [18].

CE is usually associated with rural and poor communities where practices like home slaughter, feeding dogs with offal, and disposing of dead animals without burying them are usual [4,19]. Highly endemic regions for CE are found in central Asia, Argentina, southern Brazil, Chile, Uruguay, East Africa, the Mediterranean region, and western China [20]. In 2023, Casulli et al. [21] presented an accurate evaluation of the number of cases reported in the gray and scientific literature in Europe from 1997 to 2021. A total of 64,745 CE cases were reported during that period of time. With 15,489 CE cases, Italy ranked first, followed by Spain, with 10,675 cases; Bulgaria, with 9739 cases; and Romania, with 7750 cases [21]. The World Health Organization included Romania on their list of endemic countries [22,23,24]. It has been previously documented that CE is prevalent in a number of locations in Romania [25,26]. In southern Romania, an epidemiological study carried out from 1985 to 1997 determined that the average yearly incidence of CE was 5.1 cases/100,000, with a peak incidence of 9.5 cases/100,000 inhabitants in 1997 [27,28]. Between 1991 and 2008, the CE incidence in Cluj County, northwestern Romania, ranged from 3.9 to 9.5/100,000 [29]. In two southwestern Romanian counties (Caras-Severin and Hunedoara), a mean annual incidence of 3.3/100,000 was recorded between 2004 and 2010 [28].

The current study aimed to examine and compare the demographic and epidemiological aspects of CE in children and adults hospitalized in Western Romania between 2007–2022.

## 2. Materials and Methods

This retrospective study comprised children and adults who were hospitalized with CE in Western Romania between 1 January 2007 and 1 September 2022. Data from two of our previous studies [30,31] were used to assess and compare the epidemiological findings in children and adults diagnosed with CE.

The medical charts of CE patients from four referral hospitals (County Emergency Clinical Hospital Arad, Emergency Clinical Hospital for Children “Louis Turcanu” Timisoara, “Pius Brînzeu” County Emergency Clinical Hospital Timisoara, and Municipal Emergency Clinical Hospital Timisoara) were reviewed.

Data regarding age, gender, place of residence, length of hospital stay, number of hospitalizations due to CE, cyst location, number of cysts, and occurrence of complications were collected in a database and statistically analyzed.

The diagnosis of CE was established through imaging techniques such as ultrasonography, radiography, and computed tomography. In patients who underwent surgery, CE was confirmed through anatomopathological examination.

Participants included in the study were divided into two categories according to their ages: children aged 3–17 years and adults over the age of 18 years. Individuals were subsequently separated into several subgroups: children into those aged 3–9 years and those aged 10–17 years and adults into subgroups of those aged 18–29 years, 30–39 years, 40–49 years, 50–59 years, 60–69 years, and over 70 years.

A Microsoft 365 Excel database (Microsoft Corp., Redmond, WA, USA), version 2205, was created using the collected data. The statistical analyses were carried out using the software applications EpiInfo (v.7.2, CDC, Atlanta, GA, USA, 2018) and MedCalc for Windows, version 20.015 (MedCalc Software, Ostend, Belgium). To assess the relationship between the variables, the Mantel–Haenszel test, Fisher test, *t*-test, and Chi-square test of independence were used. *p*-values less than 0.05 were considered statistically significant.

The study was conducted in accordance with the Declaration of Helsinki and approved by the Ethics Committee of Victor Babes University of Medicine and Pharmacy in Timisoara, Romania.

## 3. Results

Between 2007 and 2022, a total of 426 patients were diagnosed and hospitalized with CE in Arad County and Timis County, Western Romania. They were aged between 3 and 90 years (mean age = 41.9 ± 19.47 years). Of the total number of patients, 14.1% (60/426) were children aged 3–17 years (mean age = 11.9 ± 3.8 years), whereas 85.9% (366/426) were adults aged 18–90 years (mean age = 46.8 ± 16.2 years) (Table 1).

In 51.2% (218/426) of the cases, the patients were female, and in 48.8% (208/426), they were male. Boys accounted for 60% (36/60) of the pediatric cases, and women accounted for 53% (194/366) of the adult patients (Table 1).

Demographic data showed that 37.6% (160/426) of patients were from urban areas. Of the 41 children from rural areas, 58.5% (24/41) were boys and 41.5% (17/41) were girls, while of the 19 children from urban areas, boys accounted for 63.2% (12/19) of the cases and girls for 36.8% (7/19) (*p* = 0.78). Of the adults from rural areas, 51.6% (116/225) were women and 48.4% (109/225) were men, while in urban areas, women accounted for 55.3% (78/141) of the cases and men for 44.7% (63/141) (*p* = 0.48). No statistically significant difference was observed in terms of age, gender, or area of residence (Table 1).

**Figure 1:**
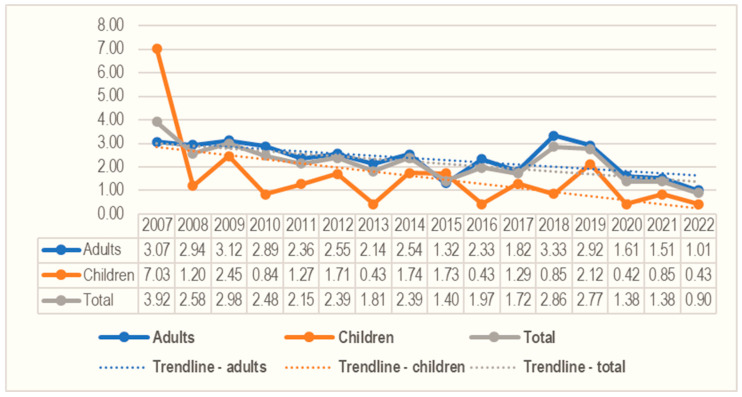
Incidence of CE in children and adults from Western Romania between 2007 and 2022.

Between 2007–2022, a descending trend in the number of cases (R^2^ = 0.45, *p* = 0.004) was noted: from 47 cases in 2007 to 11 cases in 2022. Moreover, a decreasing trend was also observed in CE incidence (R^2^ = 0.47, *p* = 0.003). The adult CE incidence decreased from 3.07/10^5^ inhabitants in 2007 to 1.01/10^5^ inhabitants in 2022 (R^2^ = 0.38, *p* = 0.009), and the incidence in children decreased from 7.03/10^5^ inhabitants in 2007 to 0.43/10^5^ inhabitants in 2022 (R^2^ = 0.27, *p* = 0.03) (Figure 1).

Most of the individuals hospitalized for CE had only one organ affected (92.3%, 393/426). The liver (81%, 345/426) was the most common location of the cyst in both children and adults, followed by the lungs (14.8%, 63/426) and various other anatomical sites (4.2%, 18/426) such as the kidney (5 cases), spleen (6 cases), peritoneum (3 cases), retroperitoneum (1 case), muscle (1 case), pancreas (1 case), and bone (1 case). Compared to cases with liver involvement, in cases with lung involvement, children accounted for a higher proportion of patients (25% with lung involvement versus 13.1% with liver involvement) (*p* = 0.02) (Table 2).

The rate of multiple-organ involvement was 7.7% (33/426) and was higher in children (16.7%, 10/60) than in adults (6.3%, 23/366) (*p* = 0.005). The results showed that 60% (6/10) of the children and 26.1% (6/23) of the adults had a liver–lung association (*p* = 0.11).

The mean length of hospital stay was 12.6 ± 7.8 days in adults and 13.3 ± 10.7 days in children (*p* = 0.54). Children with complications had a mean hospital stay of 14.4 ± 11 days, while children without complications had a mean hospital stay of 13 ± 10.7 days (*p* = 0.671). For adults with complications, the mean length of hospital stay (15.7 ± 10.7 days) was significantly longer than the mean length of hospital stay for those without complications (11.5 ± 7.3 days) (*p* < 0.001).

The majority of patients had only one hospital presentation (74.9%, 319/426). When multiple hospitalizations were evaluated, the rate was higher in children (40%, 24/60) than adults (22.7%, 83/366) (*p* = 0.004).

The rate of complications was similar between children (23.3%, 14/60) and adults (26.8%, 98/366) (*p* = 0.57). In pediatric patients, the most commonly reported complications were cyst infections (42.8%, 6/14), arrhythmia (14.3%, 2/14) and allergic reactions (14.3%, 2/14). In adults, the most-reported complications were fistulas (45/98, 45.9%), allergies (15/98, 15.3%), and cyst infections (11/98, 11.2%).

## 4. Discussion

CE is a cosmopolitan parasitic disease [32] that remains a public-health problem in many low- and lower-middle-income countries [33] and affects more than one million individuals around the world [34]. Current estimates place the annual number of new CE cases at 188,000, and the global burden of infection at 183,500 DALYs (disability-adjusted life years) [35]. In endemic regions from China, Argentina, Peru, East Africa, and Central Asia, the incidence can exceed 50/100,000 inhabitants annually [36]. *E. granulosus* is considered the second-most-significant foodborne parasite in Eastern Europe. Romania was identified as one of the countries most seriously impacted by the spread of *Echinococcus* [37].

Although people of all ages may acquire the disease, children account for approximately 10–20% of all cases [32,38]. It has been reported that in locations where the disease is prevalent in adults, the rate in children is also high [39,40]. The occurrence of CE among young people indicates that the parasite is spreading actively in the human population [4].

The overall mean age of people with CE reported in the present study is slightly lower than the mean age reported in Hungary (51.4 years old) [4]. Almost 15% of the patients in the present study were children under 18. Researchers from Hungary and Italy reported lower rates of CE in children: 1.1% and 2.3%, respectively [4,41].

In the pediatric population, boys accounted for over fifty percent of the cases. The primary reason for these gender differences is higher exposure to infection sources. Boys are more likely to help with farming and to spend more time outdoors, which increases their risk of exposure to *Echinococcus*. Boys may experience higher risk of exposure to *Echinococcus* eggs when exercising, feeding, or playing with their pets, as they are believed to be more closely bonded to dogs than girls are [39,42]. In the adult population, there was no significant imbalance in the gender distribution among cases. Reports from Palestine and Hungary identified higher numbers of CE cases in women compared to men [4,43]. The higher frequency of cases in females may be explained by rural activities mostly performed by women, including interacting with dogs, feeding dogs, cleaning up dog waste, and activities that bring individuals into contact with contaminated soil [44,45].

Most cases of illness were documented in people living in rural regions. Al-Jawabreh et al. and Amahmid et al. [39,42,43] reported results that are consistent with our findings. Two common causes of disease in rural regions are irrigating vegetables with contaminated water and using dog feces as a fertilizer [39]. Additionally, factors like relative humidity and temperature may favor the long-term resistance of parasite eggs in rural areas [39]. Therefore, a number of favorable conditions and activities make rural communities more susceptible to CE infection [39,42]. Low living standards, poor hygienic practices, lack of sanitation, and lack of knowledge of the disease are all linked to high infection rates [39]. Interestingly, more than one third of the cases involved patients from urban areas. The rates of infection were similar in children and adults. This outcome might be explained by the prevalence of stray dogs in the city suburbs. Furthermore, it has been previously noted that Romania faces a problem with stray dogs [46,47]. For children, stray dogs wandering around locations such as playgrounds increase the risk of exposure to *Echinococcus* eggs [48].

According to the current data, there was a decreasing trend in both the incidence and total number of cases in adults and children over the research period. This might be partly attributed to an increasing awareness of the disease among locals and medical professionals [49]. More rigorous safety, food, and health regulations were implemented following Romania’s 2007 EU membership [37] and the COVID-19 pandemic measures, resulting in an almost 40% decrease in hospitalizations [37]; these have also contributed to this downward trend. Between 2007 and 2016, Mustapayeva et al. also observed a decreasing trend in the incidence of CE in adults and children from Kazakhstan [49].

The majority of individuals from our study had single-organ involvement. The liver was the most common location of the cysts. These findings were similar to earlier findings that other researchers have published [34,50]. Previous studies have revealed that in pediatric CE, the lungs are affected more often than the liver is [51,52,53]. Lung-cyst development appears to be favored by the negative pressure, vascularization, and elasticity of the pulmonary tissue [9,53,54]. Despite the fact that the majority of the children in this research cohort had liver hydatid cysts, when lung involvement was examined, the rate of cases was considerably higher in children than in adults. In 3.3% of the children and 4.4% of the adults, the cysts were found in unusual locations (apart from the liver and lungs). Amahmid et al. reported unusual cyst localizations in 2.1% of the pediatric CE cases and in 6.9% of the adult CE cases from Morrocco [39,42]. It appears that adults are more likely than children are to have hydatid cysts in unusual locations [42].

Children exhibited a significantly higher prevalence of CE with multiorgan involvement compared to adults. Prior studies have indicated that up to 34.8% of pediatric CE cases involved multiple organs, with the most common association being between the liver and the lungs [55]. Recently, Mahmoudi et al. reported multiple-organ involvement in 39% of cases in children from Tehran, Iran [1]. It seems that scolexes can pass through the hepatic filter more easily in children compared to adults. This might be attributed to the lower liver-tissue density in children [56].

Children tended to have a longer mean length of hospital stay compared to adults. Among children, there was no significant difference in the mean length of hospital stay between those with complications and those without. However, in adults who experienced complications, the length of stay was considerably longer compared to those without complications (*p* < 0.001). In South Africa, children with complications had an average length of hospital stay of 9 ± 5.4 days, which was longer than the average length of hospital stay for those without complications (6.8 ± 1.5 days) (*p* < 0.001) [57]. In adults with CE from Turkey and Italy, the mean lengths of hospital stay were 5.42 ± 3.16 days and 12.1 ± 12.1 days, respectively [41,58].

The majority of the patients included in the study had a single hospital presentation. However, we noted a greater number of multiple hospital admissions in children compared to adults. In general, CE recurrence rates range from 0% to 22%. While the majority of cysts do not cause any symptoms, a recurrence may lead to serious complications such infection, rupture, or anaphylaxis. Recurrence of the hydatid cyst is the main sign of failed treatment [18]. Amahmid et al. reported no CE recurrence in hospitalized children and adults from Morrocco [39,42]. However, in pediatric CE patients from Adana, Turkey, the recorded recurrence rate was 23% [59], and in adults from Şanlıurfa, Turkey a rate of 4.5% was noted [60].

In the present study, complication rates were found to be similar between children and adults. The rate of complications in children was lower than in Addis Ababa, Ethiopia, and Constanta, Romania, where almost half of the children hospitalized with CE experienced complications. However, Thessaloniki, Greece reported a lower rate of complications in children (7.48%) compared to this study [61,62,63]. The rate of complications in adults in the present study was lower than the rate noted in Spain (34.6%) [64]. Cysts are usually detected accidentally during radiological scans or clinical examinations [65]. The underlying mechanisms of complications can be divided into three categories: infection resulting from the presence of bacteria; immunological reactions, triggered by allergic reactions to the cyst; and mechanical effects, which result from cyst compression or rupture [66,67]. The most prevalent complication in CE was identified as intrabiliary rupture of the hepatic hydatid cyst, with rates ranging from 3 to 17% [67,68]. The pericyst is avascular, meaning it does not communicate with the host’s vascular system. Thus, the rupture of the endocyst and pericyst is a necessary condition for the development of bacterial infection [67,68].

A limitation of this study is the fact that our findings are based on the data collected from patients’ medical charts. The study included only hospitalized patients, and it does not reflect the total number of CE cases in children and adults from the two counties. The real number of individuals with the disease is probably higher, but due to lack of symptoms, they might remain undiagnosed [69].

## 5. Conclusions

The present investigation provides a better understanding of the epidemiology of CE in children and adults in Western Romania. CE can be diagnosed at any age, posing a significant risk to public health in the area.

The prevalence of infection was higher in adults and children living in rural areas. The liver was the most affected organ in both children and adults, however in terms of lung involvement, children were more affected. Compared to adults, children had a higher prevalence of multiple-organ involvement. Multiple hospitalizations were also more common in children than in adults. Although the incidence and number of cases decreased in both children and adults, individuals identified with CE required hospitalization and long-term medical therapy.

This study highlights the need for new methods to stop the spread of disease. Public-health measures should be implemented and enhanced. Since most infections happen during childhood, educational campaigns on CE should be implemented in elementary schools. Unhealthy habits acquired during childhood, such as not washing hands, nail-biting, drinking water from unknown sources, and eating unwashed fruits often persist in adulthood. One of the most effective tools and strategies for achieving short- to long-term management of CE is health education. Moreover, prevention and long-term control of CE also depend on highlighting the role of contact with canids in the transmission of this zoonotic disease. Public-health measures should prioritize the management of stray dogs and promote regular deworming of household dogs.

The results of this research should be used as a basis for monitoring the evolution of CE in pediatric and adult populations. Further research should focus on age-stratified studies and address issues regarding epidemiological patterns and clinical presentation of CE cases. Age-specific risk factors, transmission mechanisms, and the socioeconomic factors that influence exposure and susceptibility in various groups should be the primary concerns of future studies. Furthermore, investigations regarding the connection between the immune system maturity, organ development, and cyst growth are essential to understand the clinical manifestations of CE in both children and adults. Filling these gaps will improve the scientific knowledge, will enhance the public health measures on CE and will result in a lower burden of the disease.

## Figures and Tables

**Table 1:** Demographic characteristics of children and adults diagnosed and hospitalized with CE in Western Romania between 2007 and 2022.

Variable		Children (%) *n* = 60	Adults (%) *n* = 360	*p*-Value
**Age group** **(years)**	3–9	16 (26.7)	-	NA
	10–17	44 (73.3)	-	
	18–29	-	70 (19.4)	
	30–39	-	60 (16.4)	
	40–49	-	72 (19.7)	
	50–59	-	83 (22.7)	
	60–69	-	46 (12.6)	
	≥70	-	35 (9.6)	
**Gender**	Female	24 (40)	194 (53)	-
	Male	36 (60)	172 (47)	0.06
**Area of residence**	Rural	41 (68.3)	225 (61.5)	-
	Urban	19 (31.7)	141 (38.5)	0.3

*p*-value: probability value. NA: not applicable.

**Table 2:** Distribution of CE cases in children and adults according to clinical characteristics.

Variable		Children (%) *n* = 60	Adults (%) *n* = 366	*p*-Value
**Number of organs affected**	**1**	50 (83.3)	343 (93.7)	**Ref.**
	**>1**	10 (16.7)	23 (6.3)	**0.005**
**Primary localization**	**Liver**	43 (71.7)	302 (82.5)	Ref.
	**Lungs**	15 (25)	48 (13.1)	**0.02**
	**Other**	2 (3.3)	16 (4.4)	1
**Length of hospital stay**	**1–7**	24 (40)	91 (24.9)	Ref.
	**8–14**	10 (16.7)	158 (43.2)	<0.001
	**>14**	26 (43.3)	117 (31.9)	0.59
**Number of hospitalizations**	**1**	36 (60)	283 (77.3)	-
	**>1**	24 (40)	83 (22.7)	0.004

## Data Availability

The original contributions presented in this study are included in the article. Further inquiries can be directed to the corresponding authors.
